# Ethical and practical challenges of generative AI in healthcare and proposed solutions: a survey

**DOI:** 10.3389/fdgth.2025.1692517

**Published:** 2025-11-17

**Authors:** Tina Tung, Shah Md Nehal Hasnaeen, Xiaopeng Zhao

**Affiliations:** 1 Department of Biomedical Engineering, University of Tennessee, Knoxville, TN, United States; 2 Bredesen Center, University of Tennessee, Knoxville, TN, United States; 3 Department of Mechanical Engineering, University of Mississippi, Oxford, MS, United States

**Keywords:** generative artificial intelligence, healthcare ethics, ethical challenges, practical challenges, large language models, bias mitigation, systematic review, solution strategies

## Abstract

**Background:**

Generative artificial intelligence (AI) is rapidly transforming healthcare, but its adoption introduces significant ethical and practical challenges. Algorithmic bias, ambiguous liability, lack of transparency, and data privacy risks can undermine patient trust and create health disparities, making their resolution critical for responsible AI integration.

**Objectives:**

This systematic review analyzes the generative AI landscape in healthcare. Our objectives were to: (1) identify AI applications and their associated ethical and practical challenges; (2) evaluate current data-centric, model-centric, and regulatory solutions; and (3) propose a framework for responsible AI deployment.

**Methods:**

Following the PRISMA 2020 statement, we conducted a systematic review of PubMed and Google Scholar for articles published between January 2020 and May 2025. A multi-stage screening process yielded 54 articles, which were analyzed using a thematic narrative synthesis.

**Results:**

Our review confirmed AI’s growing integration into medical training, research, and clinical practice. Key challenges identified include systemic bias from non-representative data, unresolved legal liability, the “black box” nature of complex models, and significant data privacy risks. Proposed solutions are multifaceted, spanning technical (e.g., explainable AI), procedural (e.g., stakeholder oversight), and regulatory strategies.

**Discussion:**

Current solutions are fragmented and face significant implementation barriers. Technical fixes are insufficient without robust governance, clear legal guidelines, and comprehensive professional education. Gaps in global regulatory harmonization and frameworks ill-suited for adaptive AI persist. A multi-layered, socio-technical approach is essential to build trust and ensure the safe, equitable, and ethical deployment of generative AI in healthcare.

**Conclusions:**

The review confirmed that generative AI has a growing integration into medical training, research, and clinical practice. Key challenges identified include systemic bias stemming from non-representative data, unresolved legal liability, the “black box” nature of complex models, and significant data privacy risks. These challenges can undermine patient trust and create health disparities. Proposed solutions are multifaceted, spanning technical (such as explainable AI), procedural (like stakeholder oversight), and regulatory strategies.

## 1 Introduction

Artificial intelligence (AI) first appeared in the 1950s; however, it was not suitable in healthcare due to its unpredictability and unexplored complexity. With deep learning (DL) coming forth in the early 2000s, AI could now learn data and use it to make its own decisions (1). From there, many have researched and produced various products that have shown promising and fast results in the field, improving predictability and understanding (1, 2). This has led to the invention of generative AI, including models like generative adversarial networks (GANs) and large language models (LLMs). The difference in generative AI, making it more appealing to medical companies, is the algorithms they possess that have the ability to generate new content based on existing data that they were trained on or that is provided to them via inputs (3). This makes them ideal for medical purposes because it allows these tools to creatively produce results using human-like thinking. With this, it also has the capability to create tailored and specialized care specific to a patient’s needs. This creates a support system for the decisions of the physicians, leading to more trust and confidence from their patient. With the correct treatments early on and remote monitoring, AI can heavily reduce patient risk and decrease the number of medical visits. In turn, this would also minimize the medical bills of patients, allowing more financially struggling patients to seek medical care. Furthermore, AI can assist in many areas through things like training enhancements, documentation reductions, clinical support, and tele-health improvements. These actions significantly improve efficiency and benefit healthcare workers in a myriad of ways. Not only that, but with the incidence of chronic diseases increasing in the U.S. to the point where 40% of adults have more than two chronic diseases with annual healthcare costs totaling about 3.3 trillion dollars, AI allows for a more rapid diagnosis and treatment process (4). This speedy process AI was able to provide especially gained interest when the COVID-19 pandemic occurred, killing over 7.1 million individuals worldwide. It has been said that, with training, AI models can be used to identify emerging pandemic threats and to assist in the research and development of a vaccine before drastic consequences occur (5).

In 2017, Arterys was approved by the U.S. Food and Drug Administration (FDA), making it the first AI-based tool to be accepted into the medical field (1). As of March 25, 2025, the list of AI/machine learning (ML)-enabled medical devices approved by the FDA has grown to 1,016 with 169 being just from the year 2024 (6). This number has increased significantly over the years. This jump started in 2016 with 18 devices approved that year. Before the year, the most devices approved by the FDA annually was 6 (7). Alongside that, the use of AI in healthcare is increasing to the point where about 75% of large organizations are planning to integrate more of these tools into faculties (8). Additionally, the global net value of AI, specifically in the medical sector, was $800 million in 2022, and it is estimated to grow to about $17.2 billion by 2032 (8). These statistics help to reveal the prevalent role generative AI will play in revolutionizing this field.

However, as these technological tools become more prevalent, a host of ethical and practical issues have emerged. The high demand for AI, coupled with its promising benefits, makes it imperative to resolve these challenges, particularly as many arise from a foundational distrust in a technology that is new and complex to many. If this distrust is not addressed, implementation may be significantly hindered. While many solutions have been developed, there remains substantial room for improvement, especially as AI tools evolve so rapidly that regulatory frameworks struggle to keep pace (9). This paper aims to provide a comprehensive overview of this landscape, from the technology’s origins to the challenges of its modern implementation. To this end, the paper is structured as follows: Section 2 provides a historical context for the evolution of AI in medicine, tracing its development from early expert systems to contemporary models. Section 3 details the systematic search methodology used for this review. The core analysis is presented in Section 4, which begins by outlining the current state of generative AI and its impact on medical training, research, and clinical practice. It then delves into the primary ethical concerns, including bias, liability, transparency, and privacy. Following this, the section critically analyzes proposed and implemented solutions and, finally, offers further suggestions to address identified gaps. The paper concludes in Section 5 with a summary of the key findings, outlining a path toward the responsible integration of AI in healthcare.

## 2 Historical context and evolution of AI in healthcare

To contextualize the contemporary ethical and practical challenges of AI in healthcare, it is essential to trace its historical evolution. The trajectory from early conceptual models to today’s sophisticated data-driven systems reveals a progressive increase in complexity, capability, and clinical integration. This historical perspective provides the necessary foundation for understanding the origins of current ethical dilemmas and for evaluating the solutions designed to address them.

The genesis of AI in medicine can be traced to the 1960s and 1970s, an era dominated by rule-based expert systems. A foundational contribution was the ELIZA program, developed in 1966 at the Massachusetts Institute of Technology. ELIZA demonstrated the possibility of natural language conversation between humans and machines, a significant milestone. It could comprehend user input containing standard sentence structures and punctuation, and generate a response (10). However, its clinical utility was limited, as the program relied on pattern recognition rather than genuine comprehension, leaving it prone to significant inaccuracies. In the same year, the invention of Shakey the Robot at the Stanford Research Institute marked a parallel advancement in robotics, creating the first autonomous agent capable of processing complex commands to plan and execute physical actions (11). By 1975, the burgeoning interest in medical AI culminated in a series of workshops hosted by the Rutgers Research Resource on Computers in Biomedicine, which served as a crucial forum for demonstrating and disseminating new prototypes and ideas (12). This period saw the development of more sophisticated expert systems, such as MYCIN in the early 1970s. MYCIN was designed to diagnose bacterial infections and recommend antibiotic treatments using a knowledge base of approximately 600 “if-then” rules derived from human experts (13). Evaluations showed its performance was comparable to that of human specialists, but it was never deployed in clinical practice due to unresolved ethical and legal concerns regarding liability for incorrect diagnoses (10). A notable advancement came in 1978 with a system for glaucoma consultation that used a causal-associational network (CASNET). This model represented a critical step beyond simple pattern matching, enabling the system to use logic and provide medical knowledge to support its outputs (14).

The 1980s witnessed the expansion and refinement of these expert systems. Models such as MYCIN, INTERNIST, and PIP became more prominent (13). INTERNIST-I, developed at the University of Pittsburgh, was particularly ambitious, aiming to cover hundreds of diseases in internal medicine. However, it struggled with the ambiguity of real-world clinical cases, especially those involving multiple comorbidities, thereby revealing the limitations and brittleness of rigid, rule-based logic (2). In 1984, a more specialized supporting model, DXplain, was developed at the University of Massachusetts. Similar to INTERNIST-I, DXplain assisted physicians by generating potential diagnoses based on patient symptoms. Its larger clinical dataset allowed for more diverse applications and enabled it to function as an early information bank, providing clinicians with access to specific details beyond immediate diagnostic support (2).

The paradigm began to shift in the 2000s with the rise of ML, DL, and the availability of massive datasets from electronic health records (EHRs). This transition marked a move away from manually coded expert knowledge toward data-driven models capable of learning patterns independently. A key milestone in this era was IBM’s Watson, which in 2007 utilized a program called DeepQA to analyze vast sources of unstructured data and generate a set of potential answers to complex questions. In healthcare, this technology expanded the scope of AI beyond simple symptom-to-diagnosis tasks, enabling more nuanced analysis of medical information (2, 15). This period also saw an explosion of DL applications in medical imaging, catalyzed by the success of Convolutional Neural Networks (CNNs) in computer vision competitions like ImageNet in 2012. This breakthrough allowed models to learn directly from pixel data, eliminating the need for manual feature extraction and dramatically improving the accuracy of image-based diagnostics.

The rapid acceleration of AI in healthcare is reflected in bibliometric trends. A study by Xie et al. found that from 1993 to 2023, publications in this field saw an average annual growth rate of 26.97%, with the most significant rise occurring between 2019 and 2023 (16). This exponential increase in research and application underscores the growing integration of AI into clinical practice and highlights the urgency of critically examining its ethical and physical requirements to ensure its responsible deployment.

## 3 Search methodology

### 3.1 Research objectives and questions

This systematic review aims to provide a comprehensive synthesis and critical analysis of the current landscape of generative artificial intelligence (AI) in healthcare. The primary objective is to systematically identify, analyze, and synthesize the existing literature on the applications, ethical and practical challenges, and proposed solutions related to the integration of generative AI into the healthcare sector. The inquiry is guided by the following research questions (RQs):

- **RQ1:** To what extent has generative AI been integrated into key healthcare domains, including medical training, research, and clinical practice, and what are the principal ethical and practical challenges (e.g., algorithmic bias, ambiguous liability, lack of transparency, and data privacy risks) that have emerged as a result?
- **RQ2:** How effective are the current data-centric, model-centric, and regulatory solutions in mitigating the identified ethical challenges, and what are their inherent limitations, practical trade-offs, and implementation gaps?
- **RQ3:** Based on the analysis of existing challenges and the limitations of current solutions, what multi-layered, socio-technical framework of governance—encompassing technical standards, organizational practices, and adaptive regulation—is required to ensure the responsible, equitable, and trustworthy deployment of generative AI in healthcare moving forward?

### 3.2 Search strategy

This systematic review was conducted in accordance with the PRISMA 2020 statement (17).

#### 3.2.1 Literature sources

We searched two electronic databases to ensure comprehensive coverage across biomedical, computer science, and general scientific literature: PubMed and Google Scholar.

#### 3.2.2 Search string formulation

A multi-tiered search strategy was developed. The foundation was a core search string combining terms for generative AI technologies with terms for the healthcare domain, formulated as follows:

(“generative artificial intelligence” OR “artificial intelligence” OR

“large language models” OR “machine learning” OR “ChatGPT”)

AND

(“healthcare” OR “medicine” OR “medical”)

To ensure depth for each thematic area, this core string was appended with additional keywords:

- *Current state of AI in healthcare:* “education” OR “training” OR “perspective” OR “telehealth” OR “application” OR “research” OR “imaging”
- *Ethical and practical concerns:* “ethics” OR “bias” OR “transparency” OR “regulation” OR “trust” OR “liability” OR “accountability” OR “malpractice” OR “privacy” OR “hallucinations”
- *Solutions:* the above terms *plus* “privacy protection” OR “cybersecurity”

All searches were limited to English-language articles published between January 2020 and May 2025 and were sorted by relevance.

### 3.3 Paper selection process

The selection of articles followed the four stages of the PRISMA 2020 model: Identification, Screening, Eligibility, and Inclusion.

#### 3.3.1 Inclusion and exclusion criteria

To be included, an article had to meet all of the following criteria:

1. Discuss applications of generative AI within a healthcare context;
2. Provide substantive coverage of either the ethical/practical challenges or the proposed/implemented solutions; and
3. Be an original research paper.

Articles were excluded if their full text was unavailable, they were published in a language other than English, or their primary focus was on non-relevant impacts such as purely financial or environmental analyses. Survey and review papers were primarily excluded, but some were included as they contained relevant information.

**Identification:** The initial database searches yielded 5,415 records. All records were imported into a reference manager for organization.

**Screening:** After deduplication, the titles and abstracts of the top 50 results per search string were screened independently by two reviewers. Records not addressing generative AI in healthcare were excluded at this stage.

**Eligibility:** The full texts of potentially relevant articles were retrieved for a detailed eligibility assessment, where the predefined inclusion and exclusion criteria were applied by two independent reviewers.

**Inclusion:** After the eligibility assessment, a final cohort of 54 articles was deemed suitable for inclusion. To ensure comprehensive coverage, the reference lists of these articles were manually reviewed in a process known as backward citation tracking, which identified additional relevant sources. Given the rapidly evolving field, the search was iterative; as novel themes emerged, targeted searches were performed to supplement the evidence base.

**Figure 1:**
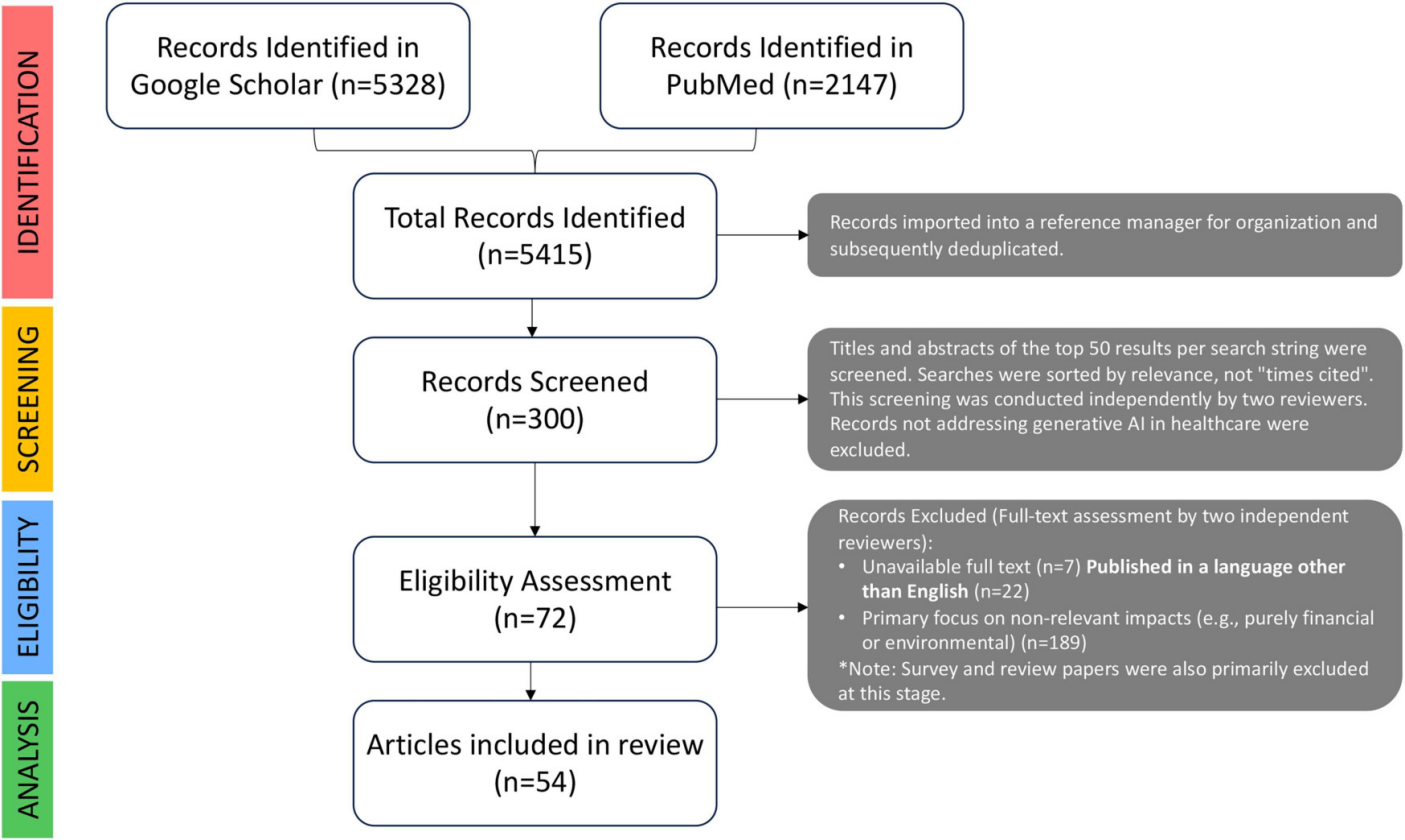
PRISMA 2020 flow diagram illustrating the identification, screening, eligibility, and inclusion of studies for the systematic survey of generative AI’s ethical and practical challenges and solutions in healthcare.

A PRISMA flow diagram summarizing this process is presented in Figure 1.

## 4 Results and discussion

For each of the 54 included articles, relevant information was systematically extracted by two independent reviewers to minimize bias. The extracted data points were organized in coherence with the research questions, and included: bibliographic details, study type, specific AI technology discussed, application domain, key findings on AI’s impact, identified ethical and practical challenges, proposed solutions, documented gaps and limitations, and authors’ recommendations for future work.

The extracted data were analyzed using a thematic narrative synthesis approach (18). This qualitative methodology was chosen for its suitability in synthesizing heterogeneous study designs, including the mix of journal articles, reviews, and surveys in this review. The process involved an initial coding of data according to themes derived from the research questions (e.g., “AI Applications,” “Ethical Challenges,” “Proposed Solutions”). This was followed by an inductive process where more granular sub-themes (e.g., specific types of bias, nuances of liability) were identified as they emerged from the data. Finally, these themes and sub-themes were woven into a coherent analytical narrative structured to directly address the research questions, exploring relationships between concepts and highlighting areas of consensus and debate in the literature.

### 4.1 Current state of AI in healthcare

This section addresses research question 1 highlighted in Section 3.1. In order to fully comprehend the ethical concerns and practical challenges, the current effects of AI in the healthcare industry will be discussed to help provide context and background. Additionally, the ways in which employees, researchers, and patients interact with the AI systems will be stated. This will show the evolution of the industry that could only be present with the incorporation of generative AI.

#### 4.1.1 The impact of AI on medical training

The training and education of medical professionals is an extensive and important process. The addition of AI has only enhanced this process to be more thorough and realistic to better prepare these individuals both mentally and emotionally.

In medical school, educators can use AI to develop in-depth curriculum content and accurate multiple choice questions for exams, granting educators more time to engage with their students (19, 20). They can also use it to discover new ways to present or teach a topic, so it is easier for students to understand. Though this is very beneficial to professors, students get the bulk of the benefits when it comes to AI. As a nonclinical learning assistant or a personal tutor, AI can help students gain knowledge in a more efficient manner (20). In addition to textbooks and course materials, ML tools can teach students by generating additional resources based on their requests and, over time, based on their weaknesses (19, 21). This includes videos, visualizations, exercises, explanations, examples, and even their own multiple choice questions for studying. Having these resources being accessible would contribute highly to the success of the student and to the understanding of the material (19). To ensure reliability, a survey-based study conducted showed that when medical students used ChatGPT to produce literature, it was well organized and much clearer than evidence-based sources (21). This study ensures that academically, these intellectual bots are helpful. To prove that they help emotionally, Li et al. performed a different study where 23 students were to engage with AI for an hour to learn more about anatomy. This study revealed a higher level of confidence for anatomical knowledge in students after talking with the chatbot. In fact, the confidence went from a previous 2.10 to a 3.84 on a scale of 5. Not to mention, these students showed a higher level of engagement, a higher performance rate in comparison to their peers, a higher sense of self-accomplishment, and a higher comfort level with making mistakes (22, 23).

On top of using AI for medical information to enhance their learning in the classroom, students can use it in a clinical setting. Having to be more hands-on can result in all kinds of little mistakes, so having a personal AI tutor would be extremely beneficial. This is especially the case with the rapid feedback they can provide on any decisions made by trainees as well as insight into their mistakes, both cognitive and practical (20, 21). In order to realistically train, doctors and trainees used to pay actors to pretend to be patients, but with the help of generative conservational AI, life-like virtual patients can be created (20, 23). Not only does this advancement allow trainees to adjust the simulation to better suit their environments, but it also gives trainers a quick and cost-effective way to go through an abundance of scenarios with their students (21, 23, 24). A study in regards to this tool has also resulted in median scores of 9 out of 10 for user-friendly and 8 out of 10 for accuracy in patient behavior with an overall 87% of participants feeling comfortable using it (23). Other forms of AI can help with training by creating simulations with artificial datasets that represent real data well but can not be linked back to any real person (24). Getting this unique experience to work with an infinite number of datasets via AI increases experience, and interacting with the virtual patients and simulations have been said to increase comfort levels (20, 21). Plus, students gain a major headstart when they use AI to train. Acclimating to regular use of AI would benefit them in the future when it becomes mainstream in the industry. Being aware of limitations in the system as well as the system itself can allow these future healthcare providers to better engage with patients and coworkers in regards to these new tools (20).

#### 4.1.2 The advancement of medical research with AI

Medical researchers spend lots of time and money on trying to improve the healthcare system. With generative AI, their work becomes more efficient. The synthetic data that AI can produce not only helps solve preexisting problems, but it also enhances the field of research in many aspects. An example of this would be using sets of artificial data as a control group. Not only does this save money and time, but it also makes it unnecessary to have a control group composed of real humans, risking their lives and privacy (24, 25). Accurate synthetic datasets can be used in studies to increase the sample size, which in turn, increases the validity and diversity of the results (24). Not to mention that this data can reduce the high fail rates experienced in managing drug discovery by testing the trial drugs in an AI system that can replicate the complexity of the human biological system rather than on animals or other humans (25).

Patient-specific models can be created from generative AI that can predict how a drug or a treatment will affect a patient based on their personal genetic profiles, and this application of AI will enable doctors to better prescribe their patients (26). Not to mention that AI and other ML models can analyze different drugs and chemicals together to predict how they will react with one another, creating a less toxic and more time efficient method for researchers to discover new drugs and doctors to prescribe current ones (26). AI is only able to do this based on its impeccable ability to recognize patterns in a large amount of data. All of this would result in a safer environment for doctors, drug developers, and most importantly, patients. To prove that point, a case study was done where an ML model was given an algorithm that allowed it to learn about all chemicals and treatments, and it was then used to come up with compounds and methods to treat people with Alzheimer’s disease (26). Using this technology to accelerate the development of drugs is essential, especially with the evolution of viruses and other harmful bacteria. Lastly, researchers could use the AI to help report their findings into organized medical writings, notably because there is predicted to be an uprise in teamwork and interactions between healthcare professionals, technological businesses, and researchers (21, 24).

#### 4.1.3 The evolution of worker responsibilities

Before the introduction of generative AI, healthcare workers experienced a heavy workload every day, and this would result in an inefficient use of materials and cost more money than needed (27). Now, LLMs and AI are used to aid in simple yet meticulous tasks, such as question answering, patient triage, and documentation writing (28–30). Alongside helping with patient health records, AI can improve the overall workflow by providing additional services, like language translation and knowledge retrieval (29, 30). This enables healthcare workers to make more time not only for their patients and any other responsibilities they may have, but also for themselves. This creates a mentally better and more productive environment for everyone.

One of the most favorable services these technologies can bring forth is the ability to support decisions made by clinicians (28–30). Rao et al. wrote about a study that involved introducing 36 vignettes to ChatGPT to test its capability on accurately diagnosing, testing, and managing patients. The results revealed an accuracy of 71.8%, showing a higher accuracy when given more clinical information. With this in mind, it can be concluded that ChatGPT does not have the ability to independently diagnose patients; however, it can assist physicians in confirming their final diagnoses (29). To further prove this point, a different study using Med-PaLM—a medical LLM created by Google—demonstrated how AI underperforms in comparison to healthcare professionals with a success rate 10.8% lower than that of clinicians (30). Another way that AI can assist in diagnosis is related to medical imaging. It can improve the quality of images by using its super-resolution algorithm to denoise, enhance, label, detect, and interpret important details (29, 31). In fact, some employers even proposed to implement a Generative Adversarial Network in their faculties, which is a system that helps resolve images (30).

#### 4.1.4 The benefits patients receive through AI

With the help of worldwide developments like reliable internet and constant smartphone use, AI has the potential to revolutionize how healthcare is provided (27). Using personal data, ChatGPT can provide medical advice on mental and physical health to improve one’s well being (21, 29). Not only that, but AI can also analyze any data from wearable devices, and even genetics, to better assist each individual (27). Arguably, the most important aspect of using generative AI in healthcare is the remote connection it can create between patients and doctors. Continuous communication and monitoring would lead to a better health outcome and faster treatment adjustments, and it would give patients a higher sense of participation in their healthcare plan, potentially causing an uprise in treatment adherence (27). To patients, money and time might be their biggest concern. Jobs can conflict with available appointment times, or money for medical bills could be tight. With the help of telehealth and AI systems like Sharesource, early interventions and remote tracking make it possible to reduce clinical visits; in fact, early interventions can help attack issues before they require routine treatments or hospitalization, saving patients money and time (27).

Though these interactive chatbots are thought to mainly evolve the world of telehealth, they also have a multitude of ways to benefit patients while in the doctor’s office. As a matter of fact, a study was done where licensed healthcare professionals were tasked to select which response they preferred—a physician’s or a chatbot’s. The results showed that they chose the chatbot’s 78.6% of the time, establishing their opinion that chatbots could help physicians create more empathetic yet informative responses to patients (30). These quick responses could lead to a happier response from patients, and it is highly convenient for both the physician and the patient when in a hurry. Another way AI is beneficial to patients is when it comes to labs, screenings, and image reviews. An example to explain this is a study assessing the role of AI in breast cancer screening. This technological tool was able to flag irregular screenings for doctors to evaluate first, enabling them to diagnose patients faster without them having to wait as long (25).

#### 4.1.5 The public perception of AI

The use of generative AI will continue to be revolutionary in the healthcare industry. Because of this, it is important to consider the opinions of those who will be interacting with this technology every day. Without keeping in mind both the positive and negative things brought forth by healthcare professionals and patients, it will be difficult to easily integrate and evolve AI in the field (32).

A study by Yousif et al. was done with medical workers to better grasp their thoughts on using AI. Doctors, pharmacists, and nurses were the primary participants. Initially, it was believed that the younger workers would be less hesitant to the idea since older workers have been traditionally doing their jobs the same way for years. Plus, older people are normally less knowledgeable with technology. This study, along with two others, has results that support this statement (32–34). More importantly, this study dives into the reasoning behind the strengths and weaknesses healthcare professionals associate with AI. To begin with the disadvantages, many fear that if these programs do improve to produce adequate answers, the workers will be replaced in the workforce either by others who understand the technology better or by the AI itself (32, 35, 36). In parallel with this is the worry of healthcare professionals becoming too over reliant and dependent on these generative answers that they do not actively think about their cases themselves, causing poor performance skills and less critical thinking without it (32, 37). A majority of the participants proceeded the study with caution due to their lack of understanding the machines’ outputs and the rationale behind them, making them question the integrity and confidence in the answers (32, 37). With no way of evaluating the accuracy and reliability of the devices due to user error, trust issues arise from many healthcare providers. These inaccuracies can lead to serious harm to patients as well as a loss of confidence in the medical worker and their abilities to make decisions (38). This hesitation can be heightened if the AI outputs something slightly reasonable but different than what the clinicians were thinking. This is supported by the survey done by Choudhury and Asan that resulted in 9% of participants displaying skepticism about the negative consequences they might experience when using AI in their clinical practice. To name the extremes of these consequences, clinicians in the survey mentioned that a false response could result in the death of a patient (39). A less extreme but still valid thought is the fact that these LLMs do not have the capabilities to deal with multisystem diseases or think creatively about patient treatments due to its training datasets not being flexible enough (38). Consequently, this also causes an absence of AI accountability from clinicians. Without the necessary requirements to fulfill the needs of healthcare professionals, there is no reason for trust to be established. Additional concerns about the patients involved inequity that may result from a lack of representation in datasets for marginalized groups as well as deficient amounts of treatment due to AI miscalculating the risk present in patients (37). Despite all this, there are still a plethora of pros that can be evaluated. On top of all the benefits already listed in an earlier section, a majority of healthcare workers showed a lot of positive thoughts about how AI can improve the healthcare industry and were willing to incorporate the use of it, even those who do not know much about its uses (32, 36, 37).

It is highly important to also consider the perception of the patients. Patients depend heavily on practitioners in regards to their health. Therefore, a solid relationship needs to be established between the two, starting with the trust of the patient. However, with the addition of AI, this doctor-patient connection could be jeopardized. Yousif et al. supported this statement by mentioning how communication gaps could result with the use of AI. This could be due to an inability for these technologies to empathize and really connect with the needs and feelings of patients (32, 40). The lack of physical touch in examinations as well as a minimal amount of patient supervision will give patients a sense of doubt and hesitation when given their diagnosis (35). In fact, a study showed that 35% of patients would decline clinicians to use generative AI in their care due to a lack of transparency and accountability as well as the risk of this new tool being inaccurate (35, 36). If patients can feel that the overall experience and quality of their medical care has deteriorated after doctors started including LLMs in their practice, they would be less likely to seek medical help when needed. A patient’s distrust in their healthcare professional can go as far as being because of a developer’s desires for personal gain. Ahmed et al. mentions that some companies can prioritize profit over patient priorities, leading to commercially built LLMs that are not fully trustworthy (38). On top of this, patients can lose trust in clinicians who primarily use AI as a solution rather than as a support system. Young clinicians who are new to the industry and are unfamiliar with patients are more likely to experience this distrust. Cestonara et al. even mentions that new physicians have an increased risk of fully trusting AI software. These potential consequences can negatively impact the motivations of future doctors (40). Both of these scenarios can lead to a reduced level of medical skills to be seen in the future. For these reasons and more, many researchers have decided to test the extent of mistrust in patients. In Robertson et al., the study conducted was specifically focused on patient “robophobia,” a term used to describe the resistance to the use of AI. To ensure validity, participants of a variety of different races and ethnic groups were chosen. Congruent with the results of other studies, this study found significant patient resistance to AI usage (41). This resistance and distrust needs to be addressed if healthcare faculties plan on incorporating AI into their clinical practices. Like the healthcare professionals, this was not enough for the majority of patients to say that the benefits do not outweigh the concerns. For example, in a study previously cited that displayed negative opinions, over 70% of patients were open to the idea of healthcare facilities using AI as long as there is valid proof that it is accurate and safe, and 60% predicted earlier diagnosis and better access to adequate healthcare with higher comfort rates (36).

### 4.2 Ethical concerns

This section addresses research question 2 highlighted in Section 3.1. While looking at the big picture, there is one category that both patients and healthcare professionals show high concerns about: ethics. Though there is some mention of ethical issues when discussing the public perception of generative AI, this section will dive deeper into the details of those concerns along with many more. Table 1 provides a summary of key literature illustrating these primary ethical challenges. Addressing these concerns not only helps tackle practical challenges, but it also plays a fundamental role in strengthening AI in the world of healthcare.

**Table 1:** Summary of AI ethical concerns.

Citations	Year	Findings	Relevance to topic
Arora and Arora (24)	2022	Patient age, sex, disease status can be extracted	Highlights confidentiality/legal issues
Rao et al. (29)	2023	Hallucinations bias AI; good at identifying meds but not doses	Shows clinical-decision risk
Choudhury and Asan (39)	2022	Workers distrust AI outputs due to liability concerns	Emphasizes trust/accountability
Vorisek et al. (42)	2023	Lack of FAIR data causes age/gender bias	Data quality impacts on AI
Cestonaro et al. (40)	2023	Opacity leads to patient distrust and clinician liability	Underlines need for transparency
Shumway and Hartman (43)	2023	Unreliable training data drives hallucinations; physicians blamed	Importance of data reliability
Le et al. (28)	2024	“Black-box” biases enter training; clinicians must infer risks	Need for model interpretability
Ahmed et al. (38)	2023	Limited training data reduces flexibility; skill gaps raise risk	Points to training requirements
Yousif et al. (32)	2024	AI’s lack of empathy causes communication gaps	Suggests empathy integration
Norori et al. (44)	2021	Cost proxies introduce racial bias in prioritization	Illustrates proxy-variable risks
Okonji et al. (45)	2024	Unclear liability fosters provider uncertainty	Calls for clear legal guidelines
Yu et al. (30)	2023	Hallucinations threaten safety; legal consequences	Emphasizes patient safety
Biswas (27)	2024	AI may widen the digital divide; needs inclusive training	Advocates broader education
Chen and Esmaeilzadeh (3)	2024	Unscreened data risks privacy breaches and hacking	Highlights security threats

#### 4.2.1 The presence of bias due to AI

In the healthcare system, LLMs have recently become the popular choice of generative AI, evolving the industry in many ways (3, 46). The algorithms of these models rely primarily on the datasets used to train them, a feature that opens up a lot of opportunities for bias if said datasets are not properly examined beforehand (3, 28). Additionally, Pal et al. found that LLMs are fragile when it comes to prompt framing and decoding parameters, so any minor changes in the parameters can alter the way the system functions. This includes making an accurate system start to hallucinate, which is a term coined to describe when AI models produce false and unreliable outputs (28–30, 47). Hallucinations can also occur due to other causes in developmental stages, making it a big concern that needs to be further researched for solutions to avoid the bias that they bring. To make matters worse, Chen and Esmaeilzadeh wrote about how adversaries have the ability to generate lots of artificial data that can be used to poison LLMs, leading to bias and tons of misinformation (3). This can negatively impact AI developers, increasing their workload and potentially damaging their reputation.

Speaking of the ability to create more datasets, sometimes researchers and trainers will use this tactic of making artificial data from already existing data as a way to further their findings or to help teach trainees (24, 48). One of the problems this illustrates is the lack of diversity in the data, limiting its ability to be realistic and to lower bias (44, 45, 48). To support the fact that this can lead to bias, a survey done by Vorisek et al. concluded that reasons for bias picked by participants were 68% because of lack of fair data and 45% because of a lack of knowledge. Additionally, this can create problems for the trainees in the future because they were trained on limited information that may not be correct representations of the general population, causing them to have some unintentional bias and to diagnose patients falsely (42, 45).

One of the main concerns when it comes to algorithmic bias is the digital divide and the increase in disparities that results. The digital divide is a barrier that forms when certain populations cannot afford to have access to these new technological tools, whether it be because of poor internet access, poor literacy skills, or poor financial situations (27). This can lead to a significant imbalance in healthcare access. Over time, this imbalance can lengthen the divide between social classes. Not only that, but those who cannot afford the machinery of AI in their healthcare facility will include to fall behind the advancements happening around them, making way for more fatalities due to the decline in quality of care. There are many other factors that can lead to an increase in bias and a decrease in equality, including gender, age, and ethnic group. There are documented examples to support this statement, such as a facial recognition example stated in Vorisek et al. that had issues recognizing female and black individuals (42). Norori et al. wrote about another example of racial bias where AI used money as a proxy to falsely determine that between a white and a black patient of equal sickness, the white patient has higher priorities because more money is spent on them in healthcare in comparison. This is even despite the fact that black populations have more severe indexes (44). With this example, it emphasizes the lack of diverse data that the LLMs are trained on. If the statistics have been proven true, those who decide on what data is used to train the systems need to account for said statistics to ensure that the AI will be accurate for those who need it most. Gender inequality can be seen in Perivolaris et al., where bias is present in mental health datasets due to underrepresentation for women as well as children, seniors, and members of the LGBTQ+ community. The result of this was faulty predictions that can lead to faulty treatment plans (48). With an unreliable treatment plan, the health of an individual can decline, and with it only occurring in particular populations, it can be seen as being purposefully. These imbalances contribute to inaccurate diagnoses while also causing further marginalization of certain populations that are already considered to be minorities. The bias does not stop at these categories; in fact, it can go as far as being biased towards words as opposed to numbers. Rao et al. did a qualitative analysis on ChatGPT’s outputs when asked for medication dosages, and the results showed that there were more dosage errors than medication errors (29). This last example of bias displays a flaw in the training data that may not have been considered when formulating the original database.

#### 4.2.2 Liability concerns for healthcare professionals

Hallucinations are one the causes of the liabilities and accountabilities that get thrusted upon medical providers with the implementation of AI. This is because the fake incorrect responses will increase doubt in physicians and their diagnoses, and if they decide to go with the decision of the AI, they are at a high risk of taking responsibility for this error (43). This can compromise the safety of patients and can have serious legal consequences for the healthcare worker (30).

There are a multitude of arguments and opinions surrounding the question of who is truly liable for a mistake when AI systems were involved: the healthcare professional or the developer. This is in part because when a nonhuman resource is used to assist in medical decisions, there is a lack of physical examination and emotional connection present that can cause curiosity to form about where the liability would lie when mistakes are made that traditionally would be backed by evidence on the patient’s body (40). An example of an opinion is written in Abràmoff et al. when they expressed that developers should be at fault if and only if the software was used properly and carefully by the provider, but if the clinician used it only in an assistive manner, then it is the fault of the clinician (49). Channa et al. supported this when they wrote that if AI is used for specialized knowledge by someone who does not have the education to be considered specialized in that field, then the liability should fall on the AI and its creator (50). Mezrich added on to the opinions by stating that the medical liability should be determined based on the degree of autonomy that was used when asking AI models for outputs. If one were to use it only as confirmation about their decision, then that person should be the one responsible for any errors (51).

The uncertainty currently present with where the accountabilities will lie creates hesitation in healthcare workers. In fact, a survey done by Choudhury and Asan showed that despite knowing the benefits, 19% of the participants were neither motivated nor willing to integrate the use of AI into their daily clinical practices because they were not prepared to have to answer for the AI’s mistakes (39). This is especially true since under the current laws that follow conventional practices, any incorrect diagnoses and unfavorable patient outcomes are legal liabilities on the provider no matter the argument (39, 45). This lack of accountability encourages clinicians to be skeptical of AI and its outputting, ultimately leading to usage refusal (39).

#### 4.2.3 Patient transparency problems

In order for generative AI to successfully enter the healthcare system, the thoughts and opinions of patients need to be taken into consideration. Too many algorithms have a lack of transparency, and this is a concern because without interpretability, it is difficult for a patient to know if the products are safe and effective (38, 52). This phenomenon of LLMs lacking these transparent capabilities is often referred to as “black box.” To expand on this, “black box” essentially means that neither the AI nor its developer can trace the process the model went through to produce its results, meaning there is no evidence to back up the accuracy of the results. This can endanger patients when false assumptions are left undetected and causes many issues with clinical oversight (28, 38, 53). Ahmed et al. even mentioned that the relationship between physicians and their patients can be negatively impacted by this lack of transparency because the clinicians would fail to explain the rationale behind how a medical device works (38).

#### 4.2.4 Privacy and safety concerns

Speaking of the rights of patients, the privacy and safety of the patient is one of the biggest priorities in healthcare. Any form of jeopardy to this right can result in major legal consequences, including the damaging of the faculty’s and the worker’s reputations and the potential risk of the patient’s life (3). Current generative AI models pose a danger to patients due to this very reason. Le et al. emphasizes this point by discussing how hallucinations can diminish the quality of patient care when practitioners choose to over rely on AI without the necessary knowledge (28). This is a reason why Biswas talked about how both patients and providers need adequate training to effectively use and understand these generative systems and to avoid misuse (27).

However, the healthcare professionals are not the only ones at fault. In fact, the LLMs themselves can threaten patient security. This is because LLMs and other generative AIs require lots of datasets to train their algorithms, which helps them to handle the extensive workload required in the healthcare industry, but it also allows a vast amount of sensitive patient information to be located in one place (3, 27). Remote patient monitoring via AI devices definitely does not help the situation with health data being constantly collected and transferred (27). Personal information—such as age, sex, disease status, and more—can be vulnerable to data breaches, unauthorized accesses, and hackings (24, 27, 54). Though using AI to generate clinical data is a very beneficial advancement in medical training, Chen and Esmaeilzadeh wrote about how hackers can target this artificial data to try and get closer to actual data, potentially leading to once anonymised data to be reidentified. (3, 24). Kim et al. goes as far as providing an example of this in the field of medical imaging. Because of the immense amounts of data in systems, unidentifiable medical images can be analyzed in such a way that reveals the original patient’s identity (55). Another way that hackers can gain access to details is by asking the LLMs themselves. If the prompts given to it are specific and clever enough, the models can be manipulated into providing confidential information (3). Hacker et al. even gave an application of what people can do with the patient details after it has been extracted. Actors can use the data of patients, or even ask an AI system to generate them fake data, and try to make insurance claims (56). All these examples can directly affect the original patients whose identity has been compromised. One example, stated in Farhud and Zokaei, can seem harmless to some patients, but it is highly inappropriate and ethically wrong. This example explores how some networks can gather data without the owner’s consent in order to boost their marketing (54). With all this in mind, it is evident that the confidentiality and safety of patients requires thorough attention when it comes to AI in healthcare.

### 4.3 Proposed and implemented solutions

This section addresses research question 3 highlighted in Section 3.1. Addressing the ethical and practical challenges of AI in healthcare requires a critical evaluation of proposed solutions, as their feasibility is often constrained by significant practical barriers and inherent trade-offs. The primary strategies—bias mitigation, transparency, liability frameworks, privacy protections, and regulation—are not independent fixes but are deeply interconnected, where the limitations of one often necessitate the implementation of another. This analysis examines the contributions of key works in each area, assessing their practical viability and overall effectiveness.

#### 4.3.1 Mitigating bias

The effort to mitigate algorithmic bias is multifaceted, involving a combination of data-centric, model-centric, and human-centric interventions. While each offers distinct advantages, none is a panacea, and their effectiveness is limited by both technical and cognitive barriers.

- **Technical and procedural interventions**
  A primary technical solution, explored by Yu et al. (30), involves the use of instruction fine-tuned LLMs. This model-centric approach trains the AI to use input for context rather than just for prediction, which is intended to produce less biased and more predictable results. The practical viability of this method is high, as it involves software-level adjustments that can be implemented by developers with relative agility. However, its effectiveness is limited because it treats the symptoms of biased data rather than the underlying cause. It risks creating a false sense of security by masking deeper data quality issues without resolving them.
  In contrast, a foundational data-centric strategy is the creation of diverse and representative datasets. Okonji et al. (45) highlights the formation of the AI for Health Imaging Initiative (AI4HI), a network aimed at creating varied databases to enhance AI training. This approach is supported by Ueda et al. (57), who identify the use of diverse datasets as one of the most efficient methods for reducing algorithmic bias. This method is highly effective as it targets the root cause of bias. Its practical viability, however, is severely hampered by the high cost of data curation, the fragmentation of medical data across institutional silos, and significant privacy hurdles imposed by regulations like HIPAA and GDPR.
  Procedural solutions focus on oversight and monitoring. Chin et al. (58) describes a multi-stakeholder collaboration involving organizations like the AHRQ and NIMHD to lower bias across the entire algorithmic life cycle. This work, along with contributions from Veluru et al. (59), proposes reactive measures such as regular audits and continuous monitoring to ensure fairness and equity over time. Ueda et al. (57) takes this further by suggesting that hospitals create dedicated departments for this purpose. The viability of these governance solutions depends on significant organizational commitment and resources. When implemented consistently, their effectiveness is high, as they provide an essential post-deployment check on model performance and can catch biases that emerge over time.
- **Human-centric interventions**
  A consensus among researchers, including Le et al. (28) and Yousif et al. (32), is that healthcare professionals must be educated to identify signs of bias in AI outputs. This human-centric solution is practical to implement through medical education and professional development. However, its effectiveness is constrained by the persistence of human cognitive biases as identified by Ueda et al. (57). Even with training, clinicians may fall prey to confirmation bias, where they are more inclined to accept AI outputs that confirm their initial judgments, thereby nullifying the benefits of a technically de-biased system. This suggests that a purely technical or educational solution is incomplete and must be part of an integrated strategy.

#### 4.3.2 Liability concerns

The ambiguity surrounding accountability for AI-related errors is a major barrier to adoption. Solutions in this area focus on professional training and the establishment of clear legal frameworks. Hale et al. (20) argues that increased training and education can help mitigate liability concerns by minimizing the chances of user error. This preventative, human-centric approach is viable but its effectiveness is limited. It addresses only the liability of the end-user (the clinician) and does not resolve questions of fault related to the AI developer or the healthcare institution, especially in cases of inherent model flaws. To address this broader issue, Ueda et al. (57) proposes the formulation of strict guidelines to formally delineate the duties and responsibilities of developers, practitioners, and healthcare establishments. This legal solution is viable in principle, but its practical effectiveness is challenged by the lack of legal precedent for AI-related malpractice cases. Without established case law, any new guidelines may be subject to multiple interpretations in court, leaving liability ambiguous.

#### 4.3.3 Transparency

A lack of transparency, or the “black box” problem, erodes trust and complicates accountability. Proposed solutions aim to improve transparency at both the clinical and technical levels. A strong consensus exists among multiple sources that clinicians must be transparent with patients about the use of AI (40, 57). This includes discussing the AI’s role, its benefits, risks, and limitations to preserve patient autonomy and enable informed consent, as emphasized by Naga Durga Srinivas Nidamanuri (25) and Biswas et al. (27). Ueda et al. (57) further specifies that patients must be made aware of how their personal data is being used, shared, and stored. This procedural and ethical solution is highly viable, as it primarily requires a shift in clinical communication practices. Its effectiveness is critical for building the foundational patient trust necessary for AI adoption.

On the technical side, Chin et al. (58) and Ueda et al. (57) advocate for the use of explainable AI (XAI) to provide evidence and understandable rationale for model outputs. To further this, Chin et al. (58) also suggests that developers can increase transparency by compiling profiles of the datasets used to train the AI algorithm. The viability of XAI is increasing as the technology matures. However, its effectiveness is limited by the inherent opacity of some complex models. Furthermore, establishing boundaries is necessary to prevent the system from becoming too transparent and leaking sensitive or proprietary information.

#### 4.3.4 Protecting privacy and safety

Protecting patient data is a foundational prerequisite for ethical AI. Solutions in this domain are a mix of manufacturer-led initiatives, organizational security measures, and employee training. Yu et al. (30) notes that some AI companies have committed to rigorous pre-release security testing to ensure compliance with safety standards. This is a viable and necessary step for reputable developers, but its effectiveness is limited to the pre-deployment phase and does not protect against threats that emerge after release.

Therefore, healthcare institutions must implement their own cybersecurity measures. Works by Veluru et al. (59), Biswas et al. (27), and Ueda et al. (57) call for strong encryption protocols, audit mechanisms, and strict access controls. Specifically, Syed et al. (60) highlights the use of multi-factor authentication (MFA) as a key tool to reduce unauthorized access. The viability of these technical solutions depends on institutional investment in modern cybersecurity infrastructure. While highly effective against many known threats, a significant limitation is that these measures are often reactive. They may be insufficient to counter proactive threats like data poisoning, where malicious data is injected during the model’s training phase. To help counter this, Veluru et al. (59) also suggests training employees on how to detect threats, adding a human-centric layer of defense.

#### 4.3.5 Building patient and worker trust

Trust from both clinicians and patients is essential for successful AI integration. To address clinician skepticism, Ayorinde et al. (37) proposes that more clear evidence is needed to demonstrate the benefits of AI in practice, along with the establishment of clear guidelines for resolving conflicts between a clinician’s judgment and an AI’s output. This approach is viable but requires significant investment in clinical validation studies and the development of robust institutional protocols. Its effectiveness is potentially high, as it directly addresses the sources of clinician distrust. To build patient trust, Ueda et al. (57) recommends involving patients and advocacy groups in the AI development and evaluation process. The viability of this co-design approach depends on the willingness of developers to engage with external stakeholders, but it is highly effective because it gives patients a voice, helps tailor the technology to their needs, and fosters a sense of shared ownership.

Finally, works by Naga Durga Srinivas Nidamanuri (25) and Le et al. (28) connect the solution of professional training directly to building trust, arguing that technologically proficient clinicians can better avoid algorithmic mistakes and more clearly explain how the AI works to their patients. This highlights the synergistic nature of these solutions. However, most current training initiatives are geared toward future medical students rather than the existing workforce, and these programs do not adequately address the risk of clinicians becoming over-reliant on AI.

#### 4.3.6 Regulating laws and guidelines

Most of the ethical concerns surrounding AI persist due to a scarcity of specific and adequate regulation. Studies by Vorisek et al. (42), Shumway et al. (43), and Farhud et al. (54) confirm that current healthcare laws are insufficient, with a survey by Vorisek et al. (42) revealing that 49% of AI specialists believe the absence of guidelines is a primary reason for algorithmic bias. This legal uncertainty is a major concern for healthcare workers, as noted by Ayorinde et al. (37). Arora et al. (24) point out a technical loophole in current laws: synthetic data generated by AI is not connected to a specific individual and is therefore not protected under existing privacy laws, creating a significant loophole. This has led to a broad call for new policies that are developed with expert input and comply with existing safety laws like HIPAA (27, 32, 57, 59).

A central critique of the current regulatory landscape, offered by Fehr et al. (52) and Palaniappan et al. (61), is the reliance on the Software as a Medical Device (SaMD) framework. The SaMD framework is ineffective for modern AI because it was designed for static software, not for adaptive algorithms that can learn and change over time. In response, “hard regulation” proposals have emerged. Shumway et al. (43) suggests mandatory pre-release testing for AI tools, while Ueda et al. (57) calls for policies requiring the public release of methodologies, datasets, and performance metrics. These proposals are viable, assuming the political will exists to enact them, but their effectiveness would depend on the rigor of the standards and the strength of the enforcement mechanisms.

A major challenge to the effectiveness of any regulatory approach is the lack of global harmonization. As detailed by Palaniappan et al. (61), Zhang et al. (62), Wang et al. (63), and Shumway et al. (43), different countries and regions are pursuing divergent paths, from the comprehensive EU AI Act to the updating of existing laws in the UK and China. This fragmentation creates complexity for developers and hinders the adoption of universal best practices. A gap noted in China’s approach by Wang et al. (63), for instance, is the lack of laws for large-scale data processing. An effective path forward likely involves a hybrid model, including legally binding “hard” principles for safety and accountability, combined with adaptive, expert-driven “soft” standards that can evolve with the technology. However, a persistent limitation in all regulatory and organizational efforts, as noted by Ayorinde et al. (37), Le et al. (28), and Hale et al. (20), is the focus on training future physicians, while often neglecting the immediate need to educate the current healthcare workforce on AI’s capabilities and limitations.

### 4.4 Further suggestions

Though there are currently many proposed and implemented solutions, there are still some gaps that need to be addressed. Table 2 synthesizes the primary ethical challenges and the limitations of current solutions, providing a clear framework for the further suggestions outlined in this section. On top of that, the world of generative AI is constantly evolving; therefore, solutions constantly need to be made to keep up. This section focuses on listing and explaining suggestions that can either address a gap or further advance a current implementation.

**Table 2:** AI challenges, current solutions, and future directions.

Concern	Solutions	Gaps	Further suggestions
Bias	∙ Use of instruction fine-tuned LLMs over foundation LLMs (30)	∙ Confirmation bias in workers	∙ Requirements for diversity in training data
	∙ Formation of AI4HI to make training databases (45)	∙ Increase in disparities	∙ Guidelines for wording AI inputs
	∙ Organizations working to lower bias at each phase of algorithmic life cycle (58)	∙ Potential hallucinations	∙ Prioritize implementation of AI in marginalized areas with support systems
	∙ Regular audits and constant monitoring of AI systems via dedicated department in hospital (57–59)		
	∙ More training for medical workers and students (28, 32)		
Liability concerns	∙ Formulation of strict guidelines regarding accountability (57)	∙ Potential copyright issues	∙ Laws addressing permission to use data from outputs
		∙ No solution for malpractice	∙ Laws with punishable actions
Transparency	∙ Benefits, risks, and limitations told to patients for them to give consent or speak up (25, 27, 40, 57)	∙ Not enough information on creation and testing of products	∙ Testing of algorithms and models with help from patients and workers
	∙ Use of explainable AI (57, 58)	∙ Products not tailored enough to needs of consumers	∙ Beta testing of models
Privacy and safety of patients	∙ Security testing before release (30)	∙ Hackers	∙ Locking AI training that cannot be accessed without special permission
	∙ Healthcare facilities investing in cybersecurity measures, strict access, encryption protocols, and audit mechanisms (27, 57, 59)	∙ Systems becoming too transparent, exposing information	
	∙ Use of MFA (60)		
Patient and worker trust	∙ Review showing evidence of AI benefits (37)	∙ Lacking in experiential training methods for more traditional workers	∙ Shadowing
	∙ Guidelines for potential disagreements between AI and workers (37)		∙ Live demonstrations
	∙ Training can help avoid mistakes, leading to more trust (25, 28)		∙ Experts alongside models to provide fast assistance
			∙ Setting on software that helps beginners with inputs
Laws and regulations	∙ AI-specific regulations to promote ethical use	∙ No standardized foundation for medical AI creation	∙ More international Laws
	∙ Policies for release of methodology and performance metrics (57)		
	∙ Many countries have created their own new laws or updated previous ones to address AI (43, 61–63)		

#### 4.4.1 Adjustments to AI manufacturing

To put an extra measure of security on their technology, manufacturers and developers should consider temporarily disabling the training of their AI models after their initial training is complete. This action essentially locks the system until it is re-opened by someone with the right authority. Other than the main developers, access to AI training should only be granted to special employees in healthcare facilities whose job is to update the algorithm. Before permission to a medical employee is granted, agreements and boundaries need to be established between them and the manufacturer. An alternative to granting access to individuals outside of the AI company is to hire specific employees who are responsible for all the AI tools present in certain healthcare institutions. The reason for this suggestion is to further protect the information of patients and to minimize the risk of hackers. On top of this, it can prevent the AI algorithm from obtaining unwarranted data and assist developers in their testing of different kinds of AI to see how they may change over time. Not to mention, it can test the diversity, explainability, and transparency of the software. Requirements for certain levels of diversity should also be enforced. Having guidelines that list all of the different populations that need to be included as well as how much of each needs to be included helps to set up a balance in the algorithm. Implementing these security measures and diversity requirements should be an immediate focus as these concerns can have large effects. Going beyond that, statistics should be regularly checked between hospitals with AI and hospitals who cannot afford it. This way, any large margins can be addresses as soon as possible to prevent long-term societal effects. As noted in a previous section, uncommon biases can arise, like being biased towards words as opposed to numbers (29). When this is noticed, thorough testing on that specific issue needs to occur. These protocols should be periodically timed with the long-term goal being that no major flaws being found. This suggestion is not as urgent as those stated before them. All in all, these recommendations work to address the ethical concern of possible hallucinations and bias that can appear in the system.

#### 4.4.2 Improvements in training

The methods to training students, as mentioned in the previous section, enable them to become knowledgeable medical professionals. The problem with this is the lack of mention of those who are already working in the healthcare industry. Though the same methods could be applicable to these workers, there is a higher chance for gaps in their knowledge. This is due to the fact that older generations are less technologically advanced and that their medical education was not taught to them alongside these new tools. Professors from universities can come give demonstrations live in the hospital to help acclimate workers, and AI developers may even sit alongside software to provide fast assistance. Though it will not help them be proficient, this solution, if implemented as soon as AI becomes a regular tool, can mitigate some technical issues. Once some medical students become proficient in the field of medical AI, it may even be beneficial for older professionals to shadow them in a sense. This way, they can experience what working with AI would be like in day-to-day life. This would be something that is considered later when students that frequently train with the LLMs enter the field. The long-term goal with this is for everyone to feel comfortable with the basic uses.

Another suggestion that can help make the implementation of generative AI smoother is for manufacturers to add an element into their models that can be turned on and off. When activated, this element would provide basic medical questions for beginner users to answer in order to recognize what information is needed for the AI to make their decision. It may hasten the process to ensure patients are receiving their care in a timely manner while simultaneously helping clinicians learn how to effectively use the models. With the element turned off, more experienced workers may ask questions in a way that would almost force the AI to give them the output they are looking for. This would increase confirmation bias. In order to combat this, it is vital that every conversation is recorded for review and that guidelines are established for the formatting of questions. Having the AI recognize incorrect formatting and expressing that in their output could also mitigate bias. These suggestions can help build the trust and knowledge of providers, which can consequently build the trust of their patients. These suggestions need to be carried out promptly, so mistrust does not have time to build within clinicians and patients.

#### 4.4.3 Consumer involvement

With patients and practitioners being the main consumers of medical AI, their opinions and experiences need to be taken into account to ensure the tools are adequately tailored to their users. One way this can be done is by performing studies with pre-diagnosed patients to find the most effective and accurate algorithm or LLM type. The participants need to cover all situations and diversities to allow for advancements in equitable care. This same study can be performed with clinicians to test the ease of use. In general, it is beneficial to perform more studies on the relationships with various AI models, patients, and healthcare professionals. This is especially true in these early stages.

Another suggestion is the use of beta testing. To clarify what this means, beta testing would involve the release of an AI tool to be used in the medical field. As flaws and improvements are found through usage, the users can provide feedback to the AI manufacturer. These companies then use the feedback to better their product, and after this is done, the product is re-released. This whole process can occur a few times, but once the major problems are fixed, the tool is finalized with the help of consumers. These suggestions help to build trust and safety between the AI companies and their customers. Additionally, it keeps the whole creation process transparent. This suggestion keeps in mind the long-term goal of making a system that works best for everyone involved.

#### 4.4.4 Further laws and regulations

Simply suggesting these changes will only lead to some individuals taking actions. Others will see it as a mere recommendation; therefore, laws need to be put into place to enforce the vital aspects of medical AI. Most countries have established new laws or updated previous ones. However, for the basic requirements, more AI-specific international laws need to be created. This way, the main foundation of all AI technology is standardized worldwide to ensure the basic rights of patients and providers are met. In order to avoid malpractice, laws that have punishable actions can be proposed. This will acknowledge the risks, raising trust in the use of AI while lessening the amount of wrongdoings. In terms of liability, it is also beneficial to establish laws regarding the permission to use the exact images and words that are outputted. This way, any incorrect words or phrases used by a healthcare worker can be traced back to the AI used, and liability can be assigned using these laws. This will also combat the issue of copyrighting. Another suggestion is for countries to ensure marginalized areas have access to AI in their healthcare systems by prioritizing technological advancements in these areas. Support systems can also be put in place to ensure that these undeserved areas are taken care of. This would mitigate disparities, supporting more equitable care. Though laws should be enforced in a timely manner, they need to be thoroughly thought out. That being considered, international laws need to be focused on immediately. This way, the differences in policies between different countries will be kept at a minimum.

## 5 Conclusion

The addition of generative AI in the healthcare industry is a process that presents many ethical and practical challenges. These obstacles need to be addressed in order for the developers, healthcare professionals, and patients to all experience the benefits that AI can offer. Examples of these challenges include the lack of transparency, trust, and regulatory laws. Bias and liability concerns are present, and patient information is at risk of being exposed. A myriad of proposed solutions are being formulated by many individuals in different countries, showing the impact that AI can make worldwide once all issues are confronted. After discussing the current state of AI in healthcare and the current concerns, this article brought forth many of these solutions as well as some implemented solutions. Limitations were identified and acknowledged, and it was further suggested that more customer involvement, training methods, and laws and restrictions are needed to help solve these challenges.

## Data Availability

The original contributions presented in the study are included in the article/Supplementary Material, further inquiries can be directed to the corresponding author.

## References

[1] Kaul V Enslin S Gross SA. History of artificial intelligence in medicine. Gastrointest Endosc. (2020) 92(4):807–12. 10.1016/j.gie.2020.06.040 32565184

[2] Hirani R Noruzi K Khuram H Hussaini AS Aifuwa EI Ely KE, et al. Artificial intelligence and healthcare: a journey through history, present innovations, and future possibilities. Life. (2024) 14(5):557. 10.3390/life14050557 38792579 PMC11122160

[3] Chen Y Esmaeilzadeh P. Generative AI in medical practice: in-depth exploration of privacy and security challenges. J Med Internet Res. (2024) 26:e53008. 10.2196/53008 38457208 PMC10960211

[4] Al Kuwaiti A Nazer K Al-Reedy A Al-Shehri S Al-Muhanna A Subbarayalu AV, et al. A review of the role of artificial intelligence in healthcare. J Pers Med. (2023) 13(6):951. 10.3390/jpm13060951 37373940 PMC10301994

[5] Suresh Gawande M Zade N Kumar P Gundewar S Weerarathna IN Verma P. The role of artificial intelligence in pandemic responses: from epidemiological modeling to vaccine development. Mol Biomed. (2025) 6(1):1. 39747786 10.1186/s43556-024-00238-3 PMC11695538

[6] Center for Devices and Radiological Health. Artificial Intelligence and Machine Learning (AI/ML)-Enabled Medical Devices. Silver Spring, MD: FDA (2025).

[7] Joshi G Jain A Araveeti S Adhikari S Garg H Bhandari M. FDA-approved artificial intelligence and machine learning (AI/ML)-enabled medical devices: an updated landscape. Electronics. (2024) 13:498. 10.3390/electronics13030498

[8] Bhuyan SS Sateesh V Mukul N Galvankar A Mahmood A Nauman M, et al. Generative artificial intelligence use in healthcare: opportunities for clinical excellence and administrative efficiency. J Med Syst. (2025) 49(1):10. 10.1007/s10916-024-02136-1 39820845 PMC11739231

[9] Zuiderwijk A Chen Y-C Salem F. Implications of the use of artificial intelligence in public governance: a systematic literature review and a research agenda. Gov Inf Q. (2021) 38(3):101577. 10.1016/j.giq.2021.101577

[10] Weizenbaum J. ELIZA—a computer program for the study of natural language communication between man and machine. Commun ACM. (1966) 9(1):36–45. 10.1145/365153.365168

[11] Kuipers B Feigenbaum EA Hart PE Nilsson NJ. Shakey: from conception to history. AI Mag. (2017) 38(1):88–103. 10.1609/aimag.v38i1.2716

[12] Kulikowski CA. An opening chapter of the first generation of artificial intelligence in medicine: the first rutgers aim workshop, June 1975. Yearb Med Inform. (2015) 10(1):227–33. 10.15265/IY-2015-016 26123911 PMC4587035

[13] Kulikowski CA. Artificial intelligence methods and systems for medical consultation. IEEE Trans Pattern Anal Mach Intell. (1980) PAMI-2(5):464–76. 10.1109/TPAMI.1980.6592368

[14] Weiss S Kulikowski CA Safir A. Glaucoma consultation by computer. Comput Biol Med. (1978) 8(1):25–40. 10.1016/0010-4825(78)90011-2 620517

[15] Ferrucci D Levas A Bagchi S Gondek D Mueller ET. Watson: beyond Jeopardy! Artif Intell. (2013) 199–200:93–105. 10.1016/j.artint.2012.06.009

[16] Xie Y Zhai Y Lu G. Evolution of artificial intelligence in healthcare: a 30-year bibliometric study. Front Med. (2024) 11:1505692. 10.3389/fmed.2024.1505692 PMC11775008 39882522

[17] Page MJ McKenzie JE Bossuyt PM Boutron I Hoffmann TC Mulrow CD. The PRISMA 2020 statement: an updated guideline for reporting systematic reviews. BMJ. (2021) 372:n71. 10.1136/bmj.n71 33782057 PMC8005924

[18] Popay J Roberts H Sowden A Petticrew M Arai L Rodgers M, et al. Guidance on the conduct of narrative synthesis in systematic reviews. In: *A Product from the ESRC Methods Programme Version*. (2006). Vol. 1. p. b92.

[19] Eysenbach G. The role of ChatGPT, generative language models, and artificial intelligence in medical education: a conversation with ChatGPT and a call for papers. JMIR Med Educ. (2023) 9:e46885. 10.2196/46885 36863937 PMC10028514

[20] Hale J Alexander S Wright ST Gilliland K. Generative AI in undergraduate medical education: a rapid review. J Med Educ Curricular Dev. (2024) 11:23821205241266697. 10.1177/23821205241266697

[21] Breeding T Martinez B Patel H Nasef H Arif H Nakayama D, et al. The utilization of ChatGPT in reshaping future medical education and learning perspectives: a curse or a blessing? Am Surg. (2024) 90(4):560–6. 10.1177/00031348231180950 37309705

[22] Li YS Lam CSN See C. Using a machine learning architecture to create an AI-powered chatbot for anatomy education. Med Sci Educ. (2021) 31(6):1729–30. 10.1007/s40670-021-01405-9 34956693 PMC8651944

[23] Sardesai N Russo P Martin J Sardesai A. Utilizing generative conversational artificial intelligence to create simulated patient encounters: a pilot study for anaesthesia training. Postgrad Med J. (2024) 100(1182):237–41. 10.1093/postmj/qgad137 38240054

[24] Arora A Arora A. Generative adversarial networks and synthetic patient data: current challenges and future perspectives. Future Healthc J. (2022) 9(2):190–3. 10.7861/fhj.2022-0013 35928184 PMC9345230

[25] Nidamanuri NDS. A study on the adoption challenges and solutions for transforming healthcare with generative AI. World J Adv Res Rev. (2022) 13(3):533–42.

[26] El-Tanani M Arman Rabbani S El-Tanani Y Matalka II Khalil IA. Bridging the gap: from petri dish to patient – advancements in translational drug discovery. Heliyon. (2025) 11(1):e41317. 10.1016/j.heliyon.2024.e41317 39811269 PMC11730937

[27] Biswas R. Innovative strategies for remote patient management in peritoneal dialysis: the role of artificial intelligence. In: *Peritoneal Dialysis in the Modern Era*. (2024). 10.5772/intechopen.1007466

[28] Le K Chang F. Intersection of AI and healthcare. JOFPCA. (2024) 2:16–18. 10.58858/010204

[29] Rao A Pang M Kim J Kamineni M Lie W Prasad AK, et al. Assessing the utility of ChatGPT throughout the entire clinical workflow: development and usability study. J Med Internet Res. (2023) 25:e48659. 10.2196/48659 37606976 PMC10481210

[30] Yu P Xu H Hu X Deng C. Leveraging generative AI and large language models: a comprehensive roadmap for healthcare integration. Healthcare. (2023) 11(20):2776. 10.3390/healthcare11202776 37893850 PMC10606429

[31] Nowakowski AZ Kaczmarek M. Artificial intelligence in IR thermal imaging and sensing for medical applications. Sensors. (2025) 25(3):891. 10.3390/s25030891 39943530 PMC11820461

[32] Yousif M Asghar S Akbar J Masood I Arshad MR Naeem J, et al. Exploring the perspectives of healthcare professionals regarding artificial intelligence; acceptance and challenges. BMC Health Serv Res. (2024) 24(1):1200. 10.1186/s12913-024-11667-9 39379939 PMC11459946

[33] Chaibi A Zaiem I. Doctor resistance of artificial intelligence in healthcare. Int J Healthc Inf Syst Inform. (2022) 17:1–13. 10.4018/IJHISI.315618

[34] Fazakarley CA Breen M Leeson P Thompson B Williamson V. Experiences of using artificial intelligence in healthcare: a qualitative study of UK clinician and key stakeholder perspectives. BMJ Open. (2023) 13(12):e076950. 10.1136/bmjopen-2023-076950 38081671 PMC10729128

[35] Moy S Irannejad M Manning SJ Farahani M Ahmed Y Gao E, et al. Patient perspectives on the use of artificial intelligence in health care. A scoping review. J Patient Cent Res Rev. (2024) 11(1):51–62. 10.17294/2330-0698.2029 PMC11000703 38596349

[36] Scott IA Carter SM Coiera E. Exploring stakeholder attitudes towards AI in clinical practice. BMJ Health Care Inform. (2021) 28(1):e100450. 10.1136/bmjhci-2021-100450 34887331 PMC8663096

[37] Ayorinde A Mensah DO Walsh J Ghosh I Ibrahim SA Hogg J, et al. Health care professionals’ experience of using AI: systematic review with narrative synthesis. J Med Internet Res. (2024) 26:e55766. 10.2196/55766 39476382 PMC11561443

[38] Imaduddin Ahmed M Spooner B Isherwood J Lane M Orrock E Dennison. A. A systematic review of the barriers to the implementation of artificial intelligence in healthcare. Cureus. (2023) 15(10):e46454. 10.7759/cureus.46454 37927664 PMC10623210

[39] Choudhury A Asan O. Impact of accountability, training, and human factors on the use of artificial intelligence in healthcare: exploring the perceptions of healthcare practitioners in the US. Hum Factors Healthc. (2022) 2:100021. 10.1016/j.hfh.2022.100021

[40] Cestonaro C Delicati A Marcante B Caenazzo L Tozzo P. Defining medical liability when artificial intelligence is applied on diagnostic algorithms: a systematic review. Front Med. (2023) 10:1305756. 10.3389/fmed.2023.1305756 PMC10711067 38089864

[41] Robertson C Woods A Bergstrand K Findley J Balser C Slepian MJ. Diverse patients’ attitudes towards artificial intelligence (AI) in diagnosis. PLOS Digit Health. (2023) 2(5):e0000237. 10.1371/journal.pdig.0000237 37205713 PMC10198520

[42] Vorisek CN Stellmach C Mayer PJ Klopfenstein SAI Bures DM Diehl A, et al. Artificial intelligence bias in health care: web-based survey. J Med Internet Res. (2023) 25:e41089. 10.2196/41089 37347528 PMC10337406

[43] Shumway DO Hartman HJ. Medical malpractice liability in large language model artificial intelligence: legal review and policy recommendations. J Osteopath Med. (2024) 124(7):287–90. 10.1515/jom-2023-0229 38295300

[44] Norori N Hu Q Aellen FM Faraci FD Tzovara A. Addressing bias in big data and AI for health care: a call for open science. Patterns. (2021) 2(10):100347. 10.1016/j.patter.2021.100347 34693373 PMC8515002

[45] Okonji OR Yunusov K Gordon B. Applications of generative AI in healthcare: algorithmic, ethical, legal and societal considerations. *TechRxiv* [Preprint]. (2024). 10.36227/techrxiv.171527587.75649430/v1

[46] Meng X Yan X Zhang K Liu D Cui X Yang Y, et al. The application of large language models in medicine: a scoping review. iScience. (2024) 27(5):109713. 10.1016/j.isci.2024.109713 38746668 PMC11091685

[47] Pal A Umapathi L Sankarasubbu M. Med-HALT: medical domain hallucination test for large language models. In: *Proceedings of the 27th Conference on Computational Natural Language Learning (CoNLL)*. (2023). p. 314–34.

[48] Perivolaris A Rueda A Parkington K Soni A Rambhatla S Samavi R, et al. Opinion: mental health research: to augment or not to augment. Front Psychiatry. (2025) 16:1539157. 10.3389/fpsyt.2025.1539157 40099144 PMC11912228

[49] Abràmoff MD Tobey D Char DS. Lessons learned about autonomous AI: finding a safe, efficacious, and ethical path through the development process. Am J Ophthalmol. (2020) 214:134–42. 10.1016/j.ajo.2020.02.022 32171769

[50] Channa R Wolf R Abramoff MD. Autonomous artificial intelligence in diabetic retinopathy: from algorithm to clinical application. J Diabetes Sci Technol. (2021) 15(3):695–8. 10.1177/1932296820909900 32126819 PMC8120059

[51] Mezrich JL. Is artificial intelligence (AI) a pipe dream? Why legal issues present significant hurdles to AI autonomy. AJR Am J Roentgenol. (2022) 219(1):152–6. 10.2214/AJR.21.27224 35138133

[52] Fehr J Citro B Malpani R Lippert C Madai VI. A trustworthy AI reality-check: the lack of transparency of artificial intelligence products in healthcare. Front Digit Health. (2024) 6:1267290. 10.3389/fdgth.2024.1267290 38455991 PMC10919164

[53] Sakamoto T Furukawa T Lami K Pham HHN Uegami W Kuroda K, A narrative review of digital pathology and artificial intelligence: focusing on lung cancer. Transl Lung Cancer Res. (2020) 9(5):2255–76. 10.21037/tlcr-20-591 33209648 PMC7653145

[54] Farhud DD Zokaei S. Ethical issues of artificial intelligence in medicine and healthcare. Iran J Public Health. (2021) 50(11):i–v. 10.18502/ijph.v50i11.7600 PMC8826344 35223619

[55] Kim B Dolz J Jodoin P-M Desrosiers C. Privacy-Net: an adversarial approach for identity-obfuscated segmentation of medical images. IEEE Trans Med Imaging. (2021) PP:1–. 10.1109/TMI.2021.3065727 33710953

[56] Hacker P Engel A Mauer M. Regulating ChatGPT and other large generative AI models. *arXiv* [Preprint]. *arXiv:2302.02337[cs]* (2023).

[57] Ueda D Kakinuma T Fujita S Kamagata K Fushimi Y Ito R, et al. Fairness of artificial intelligence in healthcare: review and recommendations. Jpn J Radiol. (2024) 42(1):3–15. 10.1007/s11604-023-01474-3 37540463 PMC10764412

[58] Chin MH Afsar-Manesh N Bierman AS Chang C Colón-Rodríguez CJ Dullabh P, et al. Guiding principles to address the impact of algorithm bias on racial and ethnic disparities in health and health care. JAMA Netw Open. (2023) 6(12):e2345050. 10.1001/jamanetworkopen.2023.45050 38100101 PMC11181958

[59] Veluru C. Impact of artificial intelligence and generative AI on healthcare: security, privacy concerns and mitigations. J Artif Intell Cloud Comput. (2024) 3:1–6. 10.47363/JAICC/2024(3)306

[60] Syed FM Kousar E S F Johnson E. AI and multi-factor authentication (MFA) in IAM for healthcare. Int J Adv Eng Technol Innov. (2023) 1(02):375–98.

[61] Palaniappan K Lin EYT Vogel S. Global regulatory frameworks for the use of artificial intelligence (AI) in the healthcare services sector. Healthcare. (2024) 12(5):562. 10.3390/healthcare12050562 38470673 PMC10930608

[62] Zhang P Kamel Boulos MN. Generative AI in medicine and healthcare: promises, opportunities and challenges. Future Internet. (2023) 15(9):286. 10.3390/fi15090286

[63] Wang C Zhang J Lassi N Zhang X. Privacy protection in using artificial intelligence for healthcare: Chinese regulation in comparative perspective. Healthcare. (2022) 10(10):1878. 10.3390/healthcare10101878 36292325 PMC9601726

